# An Erupted Dilated Odontoma: A Rare Presentation

**DOI:** 10.1155/2016/9750947

**Published:** 2016-02-16

**Authors:** Gaurav Sharma, Amritpreet Nagra, Gurkeerat Singh, Archna Nagpal, Atul Soin, Vishal Bhardwaj

**Affiliations:** ^1^Department of Oral Medicine and Radiology, Sudha Rustagi College of Dental Sciences and Research, Faridabad, Haryana 121002, India; ^2^Ahead Academy, Rajinder Nagar, New Delhi 11060, India; ^3^Department of Orthodontics, Sudha Rustagi College of Dental Sciences and Research, Faridabad, Haryana 121002, India; ^4^Department of Oral Medicine and Radiology, PDM Dental College and Research Institute, Bahadurgarh, Haryana 124507, India; ^5^Department of Prosthodontics, Kalka Dental College, Meerut 250006, India

## Abstract

A dilated odontoma is an extremely rare developmental anomaly represented as a dilatation of the crown and root as a consequence of a deep, enamel-lined invagination and is considered a severe variant of dens invaginatus. An oval shape of the tooth lacking morphological characteristics of a crown or root implies that the invagination happened in the initial stages of morphodifferentiation. Spontaneous eruption of an odontoma is a rare occurrence and the occurrence of a dilated odontoma in a supernumerary tooth is even rarer with only a few case reports documented in the English literature. We present an extremely rare case of erupted dilated odontoma occurring in the supernumerary tooth in anterior maxillary region in an 18-year-old male, which, to the best of our knowledge, is the first ever case reported in English literature.

## 1. Introduction

A dilated odontoma is an extremely rare developmental anomaly that is represented as a dilatation of the crown and root as a consequence of a deep, enamel-lined invagination and is considered a severe variation of dens invaginatus [1]. Proposed theories for origin of dilated odontoma comprise focal growth retardation theory, restricted external pressure, and focal growth stimulation in selected parts of the tooth bud [2]. Dilated odontoma originates during the morphodifferentiation stage of tooth bud formation, but its precise aetiology and genesis are unidentified [2]. Genetic factors have also been implicated as a probable factor for occurrence of dilated odontoma [2]. A dilated odontoma is however not delineated as a separate entity in the existing classification of odontogenic tumors [1]. A tooth with dilated odontoma has a circular or oval shape with a radiolucent interior and presents a single structure, often with a central soft tissue mass [3]. A dilated odontoma in the supernumerary tooth is rare and only a few cases have been reported in the recent literature [1–4]. Spontaneous eruption of an odontoma is a rare occurrence and the occurrence of a dilated odontoma in a supernumerary tooth is even rarer. We present an extremely rare case of erupted dilated odontoma occurring in the supernumerary tooth in anterior maxillary region in an 18-year-old male, which, to the best of our knowledge, is the first ever case reported in English literature.

## 2. Case Report

**Figure 1:**
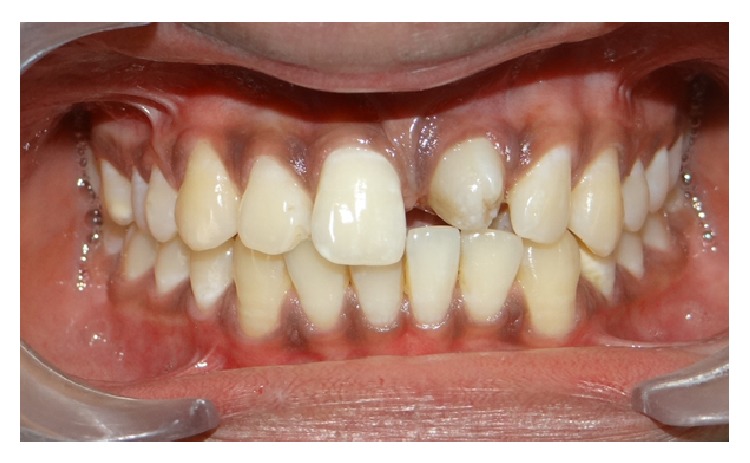
Intraoral clinical photograph depicting anomalous maxillary central incisor (frontal view).

**Figure 2:**
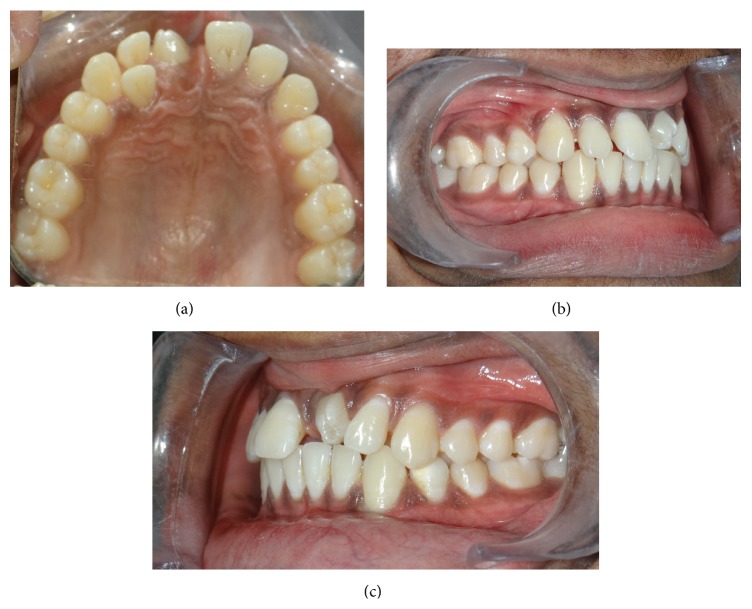
Clinical photographs of erupted dilated odontoma: (a) occlusal view, (b) right lateral view, and (c) left lateral view.

**Figure 3:**
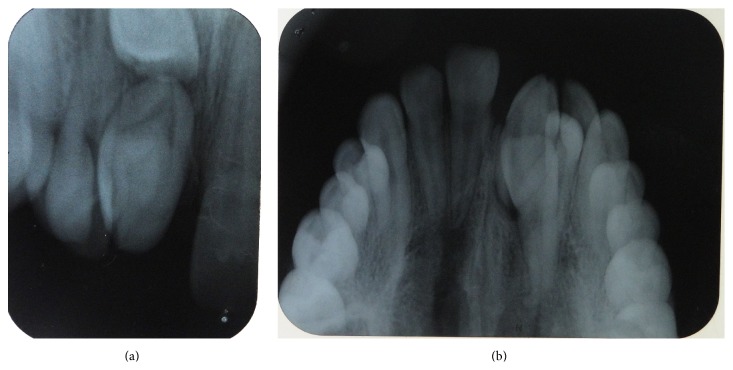
(a) Intraoral periapical radiograph and (b) anterior maxillary occlusal radiograph showing an erupted dilated odontoma with impacted central incisor.

**Figure 4:**
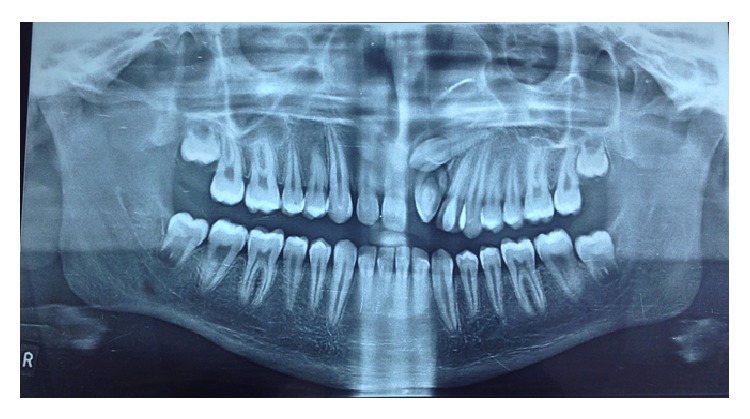
Panoramic radiograph showing erupted dilated odontoma and supernumerary incisor with impacted maxillary central incisor.

**Figure 5:**
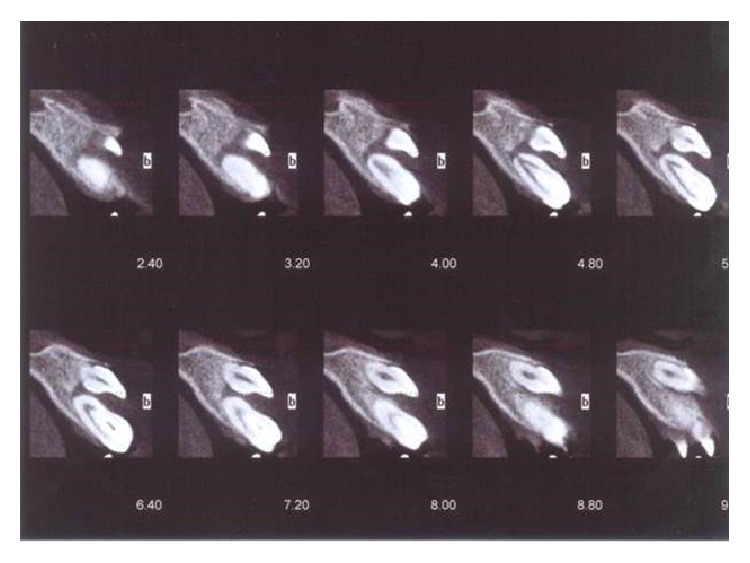
Cone beam computer imaging (CBCT) of lateral view depicting dilated odontoma.

**Figure 6:**
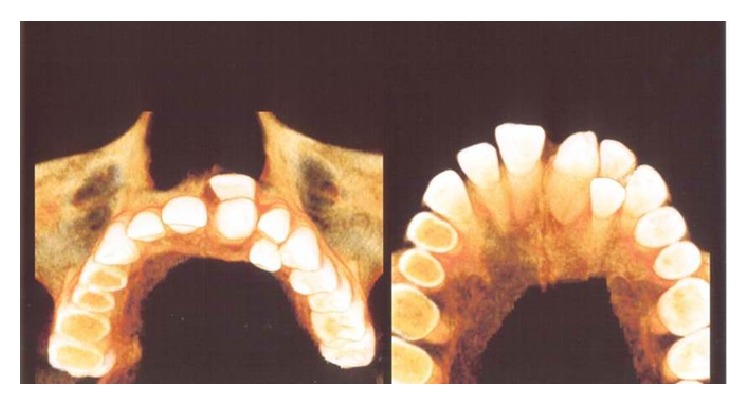
CBCT three-dimensional occlusal views depicting dilated odontoma and impacted central incisor.

An 18-year-old male presented to department of oral medicine and radiology with the chief complaint of malformed tooth in his upper front region. The patient was apparently healthy and there was no history of medical or family problem. There was no previous history of trauma to the teeth or jaws. An intraoral clinical examination revealed an anomalous tooth present in area of left maxillary central incisor (Figure 1). There was mild pain which was intermittent but no swelling or mobility was observed. The tooth did not respond to an electric pulp test and was tender on percussion. A supernumerary tooth was observed palatally in relation to permanent maxillary lateral incisor region (Figure 2(a)). The patient was advised intraoral periapical radiograph that revealed the presence of impacted permanent maxillary left central incisor (Figure 3(a)). The supernumerary tooth showed an oval radiolucent interior delineated by a well-defined radiopaque border. Patient was advised anterior maxillary occlusal radiograph that showed periapical changes in relation to anomalous supernumerary tooth (Figure 3(b)). The panoramic radiograph further confirmed the presence of impacted maxillary central incisor and supernumerary teeth in anterior maxillary region (Figure 4). Cone beam computerized tomography (CBCT) confirmed the presence of dilated odontoma (Figures 5 and 6). Based on the clinical and radiographic findings, the patient was diagnosed with dilated invaginated odontoma in supernumerary tooth in anterior maxillary region associated with periapical pathology. The diagnosis of supernumerary tooth in palatal region and impacted permanent maxillary central incisor was also done. The supernumerary tooth was subsequently extracted under local anaesthesia with written informed consent from the patient.

**Figure 7:**
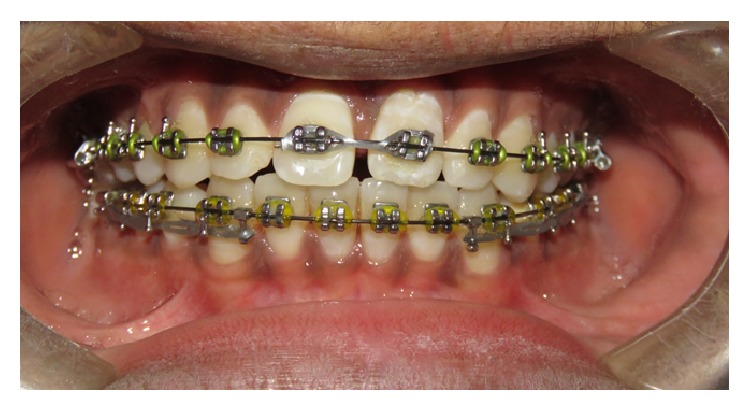
Intraoral clinical photograph (orthodontic treatment) depicting maxillary central incisor.

**Figure 8:**
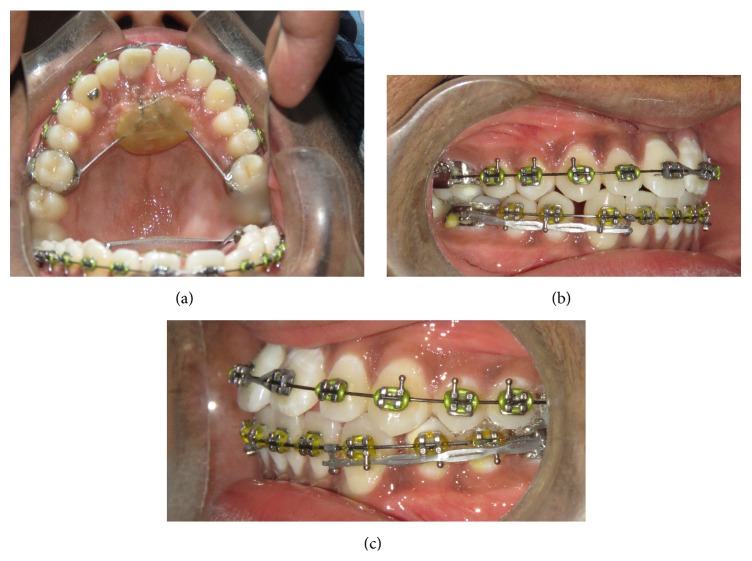
Clinical photographs of maxillary central incisor: (a) occlusal view, (b) right lateral view, and (c) left lateral view.

Impacted central incisor was surgically exposed using a full thickness mucoperiosteal flap and an attachment was bonded along with looped ligature wire on the labial surface of tooth. Flap was repositioned and a closed eruption was induced by orthodontic traction with a mild force of 60 gm, using elastomeric module. Subsequently, maxillary and mandibular arches were bonded using fixed appliance with 0.022′′ × 0.028′′ slot. Leveling of both arches was continued with sequential arch wires and space was maintained for the central incisor (Figures 7 and 8).

**Figure 9:**
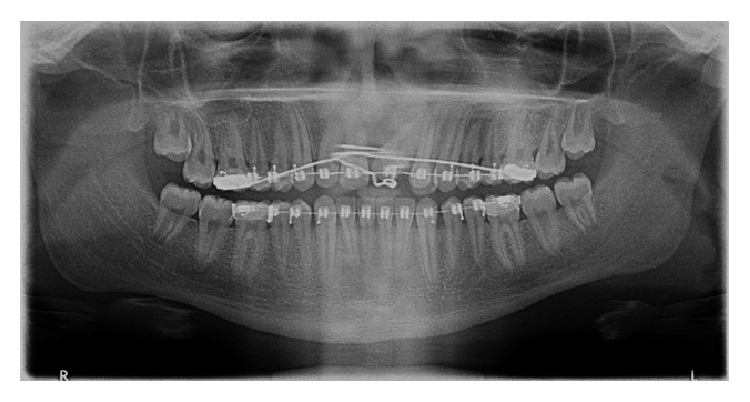
Follow-up panoramic radiograph depicting erupted maxillary central incisor.

After a period of seven months, the impacted central incisor was brought closure to the main arch; then a bracket was bonded on the labial surface of tooth and subsequently the tooth was brought into alignment with 0.012′′ NiTi as piggyback on 0.018′′ SS base arch wire. The tooth erupted and was guided into occlusion. The patient is still on follow-up (Figure 9).

## 3. Discussion

Morphological variations in dental structures involving either the crown or root have often been reported in literature and asymptomatic characteristic nature being identified only on routine radiographs. Dilated odontoma is clinically significant as there is a possibility of the early involvement of pulp [3]. An oval shape of the tooth lacking morphological characteristics of a crown or root implies that the invagination happened in the initial stages of morphodifferentiation [1]. Dens invaginatus represents a rare form of developmental anomaly with a prevalence of 0.04% with a greater female predilection [3]. The most frequently affected tooth is the maxillary lateral incisors, followed by maxillary central incisors, premolars, canines, and molars [1]. Compound odontomas have radiographic appearances reaching from solitary to multiple small tooth-like denticles in which enamel, dentin, and pulpal tissues are prearranged in a systematized pattern [5]. The most common site of compound odontoma is the anterior maxilla, characteristically over or between the roots of the erupted teeth. Though odontomas are seen frequently and constitute 22% of all odontogenic tumors, erupted odontomas are rare [5]. In the present case, the differential diagnosis of compound odontomas was excluded as they typically present with multiple rudimentary tooth-like structures.

A dilated odontoma has been described as extremely rare type of dens invaginatus [1]. Dilated odontoma is the most advanced condition of density in a tooth due to infolding of the outer tooth surface [5]. This can occur in either the crown or the root during tooth development and may involve the pulp chamber or root canal and lead to the deformity of either the crown or the root [5]. The shape is usually irregular, but the dilated varieties are often well-defined, corticated, round, or oval masses with radiolucent centres. In our case, the patient presented with a deformed crown with an oval shaped radiolucency suggestive of dilated odontoma.

Coronal dens invaginatus has been categorized in 3 groups by Oehlers based on radiographic interpretation according to the degree of invagination. In type I Oehlers classification the enamel lined invagination ends as a blind sac within the crown and is not ranging beyond the cement-enamel junction (CEJ). Type II Oehlers classification represents the enamel-lined invagination extending apically beyond the CEJ but persists within the root. In rare type III Oehlers classification, the enamel-lined invagination extends apically beyond the CEJ and communicates laterally with the periodontal ligament space with no involvement of the pulp [1].

The possibility of the erupted tooth being supernumerary was considered in the current case as the impacted tooth was anatomically similar to the permanent central incisor on a radiographic examination. The above case is unique as the patient also had hyperdontia and there was the presence of a dilated odontoma in an erupted tooth in anterior maxillary region. However, there is no description of an erupted dilated odontoma [6, 7]. Most of dilated odontoma cases have been reported from the posterior mandibular areas and have been impacted frequently. Recently a case report of the occurrence of bilateral odontomas in anterior maxillary teeth with hypodontia and peg laterals was observed [8]. In our case there was a triad of dilated odontoma, supernumerary supplemental incisor, and impacted maxillary central incisor. The authors suggest that the dilated odontomas in concurrence with other dental anomalies should be further investigated for a genetic analysis as it might represent a chromosomal trait.

The association of dens invaginatus with supernumerary tooth is a very rare phenomenon. Extensive PubMed search revealed that only six case reports have been published in English literature till now [9]. However all the cases were of dens invaginatus of milder variety and none of the cases had documented the occurrence of dilated odontoma in a supernumerary tooth. To the best of the authors' knowledge, the present case represents the first case report of an erupted dilated odontoma in a supernumerary tooth. The occurrence of supplemental maxillary incisors is much less common than conical or tuberculate supernumerary teeth. Supplementary maxillary lateral incisor is however more commonly observed as compared to a supplemental maxillary central incisor [8]. In the present case, a supplemental maxillary central incisor was observed palatally. Chronic inflammation at the apical region of the dens invaginatus can lead to a condition called periapical invaginitis [10]. The invagination repeatedly leads to the access of irritants promptly into the pulpal chamber due to a thin permeable membrane [3].

In case of an exceedingly unusual anatomy such as the current case, surgical or endodontic management is not practicable, and extraction is favoured as a last choice of treatment [9]. Amalgamating additional imaging modalities with a comprehensive clinical examination and history is required to achieve an adequate diagnosis and for subsequent management [11]. Extraction was considered in our case in order to facilitate the eruption of the permanent maxillary central incisor in conjunction with orthodontic management. A multidisciplinary approach comprising an oral radiologist, oral maxillofacial surgeon, and orthodontist should be the treatment of choice for these rare morphological variations as an early diagnosis and treatment are very important to prevent physiological, aesthetic, and functional problems.
